# The prevalence and associated factors of birth asphyxia among neonates delivered in Public Hospitals, Northern Ethiopia

**DOI:** 10.4314/ahs.v22i2.60

**Published:** 2022-06

**Authors:** Selamu Abose, Amanuel Nuramo, Merhawi Brehane, Lire Lemma, Ritbano Ahemed, Haftom Gebrehiwot

**Affiliations:** 1 Department of midwifery, College of Health science, Wachemo University, Ethiopia; 2 Department of midwifery, College of Health science, Mekelle University, Ethiopia; 3 Department of Public Health, College of Health science, Wachemo University, Ethiopia

**Keywords:** Birth asphyxia, associated factors, Neonates, Northern Ethiopia

## Abstract

**Introduction:**

A body of evidences showed that birth asphyxia is a serious public health problem in low income countries including Ethiopia. There are sparse data on the prevalence of birth asphyxia and its associated factors among neonates in low income countries like Ethiopia, as well as the research area.

**Objective:**

Therefore, this study determined the prevalence and associated factors of birth asphyxia among newborns administered in public hospitals in Northern Ethiopia, 2019.

**Methods and materials:**

A cross-sectional study of health institution was carried out in December 2019. Systematic sampling technique was used. Data was collected through interviews and chart review. Multivariate logistical regression analysis was done to control confounders and identify significantly associated variable. AOR with 95% confidence intervals were computed to identify the factors independently assoiated with birth asphyxia.

**Results:**

The finding showed that the prevalence of birth asphyxia was 20.0%. Induction of labor (AOR=3.59, 95% CI: 1.36–9.46), Prolonged labor (AOR=3.59, 95% CI: 1.36–9.46), meconium-stained amniotic fluid (AOR=3.49), referred mothers (AOR=3.68, 95 % CI: 1.46–9.28), instrumental delivery (AOR=2.87, 95% CI: 1.09–7.55)and primiparous mothers (AOR=2.048 95% CI: 1.10–3.80). were significantly associated with birth asphyxia.

**Conclusion:**

The Prevalence of birth asphyxia notable high. Therefore; intra-partum care services should be strengthened to prevent birth asphyxia.

## Introduction

Birth asphyxia is a serious clinical problem worldwide^1^,^2^. The World Health Organization (WHO) estimated that 3% of approximately 120 million infants born in low income countries develop birth asphyxia^3^. The WHO (2016) defines birth asphyxia as a failure to initiate and sustain breathing at birth^4^.

The most common criteria for diagnosing asphyxiation at birth in developing countries is the Apgar score. In addition, clinicians use the history, physical examination, and lab tests for accurate diagnosis^5^

Although it is a global health issue, the extent of asphyxia at birth varies between low income and high income countries because of the difference in care and availability of resources^6^. The rate was 1/1000 live births in high income countries, compared with 5–10/1000 live births in low income countries^7^. It was highest in Africa (27%), followed by the Eastern Mediterranean (26.6%), Southeast Asia (24.3%), the Americas (7.7%), the Western Pacific (6.7%) and lowest in Europe^8^.

Asphyxiation at birth results in a wide range of outcomes, such as neurological disorders and emotional impairments^9^. According to the 2016 WHO report, it represented 33.2% of all impairments^10^. Early intervention for 10% of at-risk mothers would save about £20 million per year^11^. In Nigeria, asphyxia at birth contributed to 12% of neonatal intensive care admissions, 24% of neonatal deaths, 5.3% of these cases suffered multiple organ failure^12^. In a study in Addis Ababa, Ethiopia, 16.1% of newborns were admitted and 40% died^13^. In Ethiopia, over all pooled estimated prevalence from six studies^14^ was 24.06 %.

In the last two decades, the Government of Ethiopia has implemented the health sector development program to improve access and quality of health services to all segments of the population^15^.

A body of evidence has shown that birth asphyxia is a serious public health problem in low income countries, including Ethiopia^16^, ^17^, ^19^, ^24^, ^21^, ^24^. The neonatal period is the last chance to suffer from asphyxia at birth. Despite this, asphyxiation at birth was found to be a low risk of neonatal complications.

There are sparse data on the prevalence of birth asphyxia and its associated factors among neonates in low income countries like Ethiopia as well as research area^19^,^20^,^21^,^24^ And it is difficult to extrapolate the literature from higher income countries to low income countries and also region to region where ecological differences, low antenatal care services and higher rate of perinatal morbidity and mortality complicates the comparison. Identifying the prevalence of birth asphyxia and associated factors helps to design appropriate intervention by the health institutions, communities and organizations to combat the problems. The findings of this study will contribute to the body of knowledge and help for obstetric health care providers and other stakeholders who work on maternal and child health to formulate relevant and practical measures to tackle this challenge ;and, finally to decrease newborn morbidity and mortality. Therefore, this study determined the prevalence and factors associated with birth asphyxia in public hospitals of northern Ethiopia, 2019

## Methods and materials

### Study period and area

The study was done in Mekelle's public hospitals from 1 to 30 December 2019. Mekelle is the capital city of Tigray regional state. It is located 730 km north of Addis Ababa. According to central statistical agency the total population of Mekelle is 483,217 (CSA, 2015). It has one comprehensive referral Hospital, 3 general Hospitals and 9 health centres.

### Study design

A cross-sectional study of health institutions was carried out.

### Source population

All newborn babies with or without birth asphyxia delivered in the public hospitals in Mekelle city, 2019.

### Study population

Selected newborn babies with or without birth asphyxia delivered in the public hospitals in Mekelle city, 2019

### Sample Size Determination

The sample size was calculated using a single population proportion formula based on the proportion of asphyxia at the birth of a study in the Jimma of Oromia, Ethiopia18. Assuming a 5% margin of error and a 95% confidence level and adding 10% of the non-response rate, the total sample size was 371.

### Sampling technique and procedure

Systematic random sampling was used to select mothers who are born alive. The sampling was started by selecting the first mother and continued every k. K was calculated by taking average month delivery of public hospitals in Mekelle city. The average month delivery was calculated from the total annually delivery of public hospitals of Mekelle.

### Operational Definitions

Incomplete ANC visits- Mother with 1–3 ANC visitors. Birth asphyxia- Apgar scores below 7 in the first minute.

### Data Collection Tools and data collection procedures

A structured questionnaire was prepared and used following a review of relevant documents. The questionnaires focused on socio-demographic, prenatal, intrapartum and neonatal characteristics. Questionnaires prepared by the English, then translated into the local language Tigrigna to check its coherence and again translated back the English. Data were collected through interviews with mothers and file reviews. Data were collected by six BSc Midwives and two MSc Midwives supervised them.

### Data quality control

In order to guarantee data quality, training has been provided to data collectors. The Principal Investigator and the Supervisor verified the completeness, clarity and consistency of the data collected immediately at the end of each data collection day. The pre-test verified 5% (18 samples) at the Wukoro General Hospital and helped reorganize the order of the tools.

### Data processing and analysis

The data was checked, coded and entered in epi-data version 4.2 and exported to SPSS version 20 for analysis. Descriptive data was presented by table and text. A bivariate logistic regression was carried out to examine the association of the dependent variable with each independent variable. A P-value 0.2 was used to consider candidate variables for multivariate analysis. Multivariate logistical regression analysis was done to control confounders and identify significantly associated variables. Adjusted Odds ratio with 95% confidence intervals was computed to identify the presence and strength of association A p-value <0.05 was used to identify variables significantly associated with asphyxiation at birth.

## Results

### Socio-demographic characteristics of mothers

A total of 371 mothers and their neonates were included in the study with a response rate of 100%. The median age of the mothers was 26 years and about one-third (34.2%) of the mothers were in the age group of 21–25 year. Approximately one-third (76.3 per cent) of mothers were urban residents. Over one quarter (27.2%) of mothers attained to higher education. Concerning the religion and ethnicity of mothers, 86% of were Orthodox by religion and 88.9% were by ethnicity. Most of the mother's income was greater than the median (Table 1).

**Table 1:** Socio-demographic characteristics of the mothers in public hospital of Mekelle city, Northern Ethiopia, 2019(n=371)

Variables	Category	Frequency (%)	Percentage (%)
Age in years			
	15–20	37	10
	21–25	128	34.5
	26–30	120	32.3
	31–35	57	15.4
	>=36	29	7.8
Religion			
	Orthodox	322	86.8
	Muslim	43	11.4
	Protestant	6	1.6
	Tigray	361	97.3
	Others	10	2.7
Marital status			
	Married	350	94.3
	Unmarried	21	5.7
Residence			
	Urban	283	76.3
	Rural	88	23.7
Occupation			
	Employer	121	32.6
	Merchant	77	20.8
	Housewife	132	35.6
	Farmer	41	11..1
Mother education			
	Non-educated	34	9.2
	Primary	101	27.2
	Secondary	123	33.3
	Higher education	113	30..2
Husband education			
	Non-educated	9	2.6
	Primary	63	18.1
	Secondary	110	31.2
	Higher education	166	47.7
Income of mother			
	<2000	117	31.5
	≥2000	254	68.5
Total n(%)		371	100

### Characteristics of Neonates

Among 371 newborns, (97.5%) were single, (96.7%) had normal birth weight and (97.5%) had no gross congenital abnormalities. (Table: 2). Over half of the neonates (53.3%) were male. The ratio of newborns to men was 1.1:1, and asphyxiation at birth was 20.0%.

**Table 2:** Characteristics of neonates in public hospitals of Mekelle, Northern Ethiopia, 2019(n=371)

Variables	Category	Frequency	Percentage
Sex of newborn	Male	199	53.6
	Female	172	46.4
Birth weight			
	Normal weight	352	94.9
	Low birth weight	14	3.80
	Macrosomia	5	1.35
Number of gestation			
	Single	362	97.5
	Multiple	9	2.5
Gross congenital abnormality	No	362	97.5
	Yes	9	2.5
	Preterm	45	12.1
	Term	303	81.7
Gestational age	Post term	23	6.2
Asphyxia at first minute	No	297	80.0
	Yes	74	20.0
Asphyxia at 5^th^ minute	No	339	91.3
	Yes	32	8.7
Total n (%)		371	100

### Obstetric characteristics of the mothers

More than half of them were multiparas (52.7%). Among multiparous mothers; 7% have a history of abortion and 5.8 % neonatal deaths, and among 371 mothers, 85.4% of mothers give birth within 24 hours of the onset of childbirth. Midwives accounted for 85.4% of births (Table: 3).

**Table 3:** Obstetrics Characteristics of mothers in public hospitals Mekelle, Northern Ethiopia, 2019(n=371)

Variables	Category	Frequency	Percentage
History of abortion			
	No	345	93.0
	Yes	26	7.0
Parity	Primipara	177	47.7
	Multipara	194	52.3
History of still birth			
	No	175	90.2
	Yes	19	9.8
History of neonatal death	No	183	94.3
	Yes	11	5.7
Inter-delivery interval			
	≥2 years	164	84.5
	<2 years	30	15.5
Number of ANC visit			
	No	5	1.3
	1–3 visit	67	18.0
	>=4 visit	299	80.70
Pregnancy desire			
	Planned	344	91.6
	Not planned	31	8.4
Alcohol drinking			
	No	362	97.6
	Yes	9	2.4
Pregnancy induced hypertension	No	346	93.3
	Yes	25	6.7
Premature rupture of membrane	No	347	93.5
	Yes	24	6.5
Anemia			
	No	365	98.4
	Yes	6	1.6
Poly-hydrmnios*			
	No	365	98.4
	Yes	6	1.6
Oligohydraminous			
	No	357	96.2
	Yes	14	3.8
Antepartum hemorrhage			
	No	352	95.1
	Yes	19	4.9
Febrile illness	No	366	98.6
	Yes	5	1.4
Duration of labor			
	<24 hour	317	85.4
	≥24 hour	54	14.6
Augmentation			
	No	356	95.9
	Yes	15	4.1
Feto-pelvic disproportion	No	359	96.8
	Yes	12	3.20

### Factors associated with birth asphyxia

Parity, mode of delivery, onset of labor, duration of labor, amniotic fluid level and reserved status were significant factors associated with asphyxia at birth (Table 4). Newborns of primiparous mothers were 2 times more likely to be asphyxiated than newborns of multiparous mothers (AOR=2.048 95% CI: 1.10–3.80).

**Table 4:** Multivariate logistic regression analysis of factors associated with birth asphyxia in Public Hospitals of Mekelle city, Northern Ethiopia, 2019

Variable	Category	Cross tab(Birth Asphyxia)				
		No n (%)	Yes n (%)	COR (95%CI)	AOR	P-value
**Parity**	Primi Para	130(73.4%)	47(26.6%)	**2.23(1.32–3.78)**	**2.04(1.10–3.80)** *	**0.024** *
	Multipara	167(86.1%	27(13.9	1		1
**Pregnancy desire**						
	Planned	276(81.2%	64(18.8%)	1		1
	Not planned	21(67.7%)	10(32.3%)	2.05(0.92–4.57)	1.54(0.54–4.34)	0.40
**Gestational age**	Term	249(82.2%)	54(17.8%)	1		1
	Preterm	31(68.9%)	14(31.1%)	0.614(0.23–0.63)	1.79(0.74–4.29)	0.20
	Post term	17(73.9%)	6(26.1%)	1.28(0.416–3.939	1.00(0.29–3.43)	0.99
**Oligo-hydraminous**						
	No	289(81.0%)	68(19.0%)	1		1
	Yes	8(57.1%)	6(42.9%)	3.18(1.07–9.49)	2.12(0.480–9.42 )	0.320
**Mode of delivery**	SVD	256(87.1%	38(12.9%)	1		1
	Instrumental delivery	15(51.7%)	14(48.3%)	6.28(2.8–14.1)	**2.87(1.09–7.55)** *	**0.033** *
	Cesarean delivery	26(54.2%)	22(45.8%)	5.70(2.94–11.05	1.658(0.607–4.530)	0.321
**Onset of labor**						
	Spontaneous	284(82.8%)	59(17.2%)	1		1
	Induction	12(44.4%)	15(55.6%)	6.01(2.67–13.5)	**3.59(1.36–9.46)** *	**0.01** *
**Duration of labor**						
	<24 hour	266(83.9%)	51(16.1%)	1		1
	≥24 hour	31(57.4%)	23(42.6%)	3.870(2.08–7.17)	**2.781(1.32–5.85)** *	**0.007** *
**Amniotic fluid** **status**						
	Clear	269(84.1%)	51(15.9%)	1		1
	Bloody stained	11(61.1%)	7(38.9% )	3.357(1.250.067)	1.481(.408–5.377)	0.550
	Meconium stained	17(51.5%)	16(48.5%)	4.964(2.35–610)	**3.491(1.430–8.524)** *	**0.006** *
**Birth** **Attendant**						
	Midwife	269((84.9%)	48(15.1%)	5.204(2.81–9.63)	2.419(0.979–5.93)	0.056
	Doctor	28(51.9%)	26(48.1%)	1		1
**Booked status**						
	Not-referred	285(83.8%)	55(16.2%)	1		1
	Referred	12(38.7%)	19(61.3%)	8.20(3.76–17.86)	**3.687(1.463–9.288)** *	**0.006** *

* p<0.05(significantly asso ciated factor)

1=reference

SVD=spontaneous vaginal delivery

Neonates who were born by induced labour were 3.6 times more likely to be asphyxiated as compared to neonates born by spontaneous labour (AOR=3.59, 95% CI :1.36–9.46).

The chances of asphyxiation at birth were 2.78 times higher among newborns born in prolonged labour than those born less than 24 hours. (AOR=2.78; 95% CI: 1.32, 5.85).

Neonates who were delivered by instrument (forceps and vacuum) had more likely to have birth asphyxia as compare to those neonates who were delivered spontaneously (AOR=2.87, 95% CI: 1.09–7.55).

The odds of the asphyxia were 3.68 more for neonates of referred mothers compared to the neonates of non-referred mothers (AOR=3.68, 95 % CI: 1.46–9.28) The odds of asphyxia were 3.08 times higher for neonates whose mothers had meconium stained amniotic fluid compared to neonates of the mothers with clear amniotic fluid (AOR=3.49, 95% CI: 1.43–8.52).

## Discussion

The prevalence of birth asphyxia is nearly similar to with the study in Ethiopia 24. It is lower than the finding in a study done in Bangladesh 22, Kenya 23 and Ethiopia. This difference from the study in Bangladesh might be due difference in the sampling technique. This differs from a study done in Jimma could be the difference in characteristics of participants such as ANC coverage, medical complications and obstetric complications. In addition the study I in Jimma included very preterm neonates. In general, the differences in the magnitude of birth asphyxia among these studies are the difference between the characteristics of mothers and neonates, methods and the setting.

Regarding parity, neonates from primiparous mothers had higher odds of birth asphyxia than multiparous mothers. A similar finding was revealed in Bangladeshi^22^, Kenya 23 and Uganda 13. This could be the possibility that primiparous mothers could have inadequate uterine contraction (hypotonic uterine contraction) which results in prolonged labor. This prolonged labor has the possibility of maternal exhaustion and non-reassuring fetal heart rate which might result in birth asphyxia. Another possibility could be lack of experience and poor cooperation during labour may leads to birth asphyxia among primiparous mothers. Disagree in the study done in Ethiopia 19, 20. This could be a difference in the methods and characteristics of participants.

Neonates delivered by instrument had more odds of having birth asphyxia as compare to those neonates delivered spontaneously. This finding agreed with the study done in Pakistan 3. This could be due the possibility of instrumental delivery leading shoulder dystocia, which might have resulted in hypoxia and finally resulted in birth asphyxia^26^, ^27^. Another possibility may be the condition of neonate during intrapartum period.

Neonates who were born by induction were more likely to have asphyxia as compared to neonates of mothers who started labor spontaneously. This finding agreed with studies Kenya 23. The possible reason could be the titanic contraction of the uterus (more than normal duration and frequency) during induction of labor, which could lead to cord compression that might result in asphyxia. Another possibility could be rapid birth during the induction of labor, which results in birth trauma, which results in birth asphyxia. The third possible reason could be a high resting tone during induced labor than spontaneous labor.

Prolonged labor had a positive association with birth asphyxia. This finding is similar to the study done in Ethiopia 23, and Kenya 22. This could be due to high-energy utilization and dehydration in mothers with prolonged labor might result in birth asphyxia^25^, ^26^. Another possible reason could be due to the management of prolonged labor, which may cause brain damage and anesthesia effect and finally may lead to birth asphyxia. The third reason might be prolonged labor could be due to Cephalo-pelvic disproportion. This might leads to brain damage due to bleeding in the brain during labor. Moreover, this could lead to birth asphyxia.

The odds of birth asphyxia were higher for neonates whose mothers had meconium-stained amniotic fluid as compared to neonates of the mothers with clear amniotic fluid. The finding is supported by the study in Bangladesh^22^ and in Ethiopia 24. This could be due to the possibility of aspiration of meconium when the newborn breathes in during delivery, which could result in difficulty of fluid exit and alveolar collapse and made the difficulty of breathing 25.

The odds of the birth asphyxia were more for neonates of referred mothers as compared to the neonates of the non-referred mothers. This could be due to the possibility of delay in receiving care, which might have resulted from the delay in seeking care, transportation, and health facility. However, the referral status of mothers had no association with birth asphyxia in the study in Kenya 23. This could be due to difference in setting and resource availability

## Conclusion

The prevalence of birth asphyxia is high compared to the expected frequency of birth asphyxia in low income countries. Parity, onset of labour, duration of labour, booked status, mode of delivery, and amniotic fluid status were factors significantly associated with birth asphyxia. All these factors are preventable. Therefore, there is needed for Zonal health department of Mekelle strengthens intra-partum care services for prevention of birth asphyxia through prevention of delays. Hospitals and health professionals should strengthen close monitoring of labour with the partograph; Timely communication and are critical.
